# Endemic Vascular Epiphytes: Integrating Protected Areas and Suitability Models in the Amazon Forest

**DOI:** 10.1002/ece3.72407

**Published:** 2025-11-29

**Authors:** Keller Maurício dos Santos Lucas, Adriano Quaresma, Giuliette Barbosa Mano, Maria Teresa Fernandez Piedade, Viviane Pagnussat Klein, Layon Oreste Demarchi, Jochen Schöngart, Aline Lopes

**Affiliations:** ^1^ Ecology, Monitoring and Sustainable Use of Wetlands Research Group (MAUA) Instituto Nacional de Pesquisas da Amazônia (INPA) Manaus Brazil; ^2^ Department of Wetland Ecology, Institute of Geography and Geoecology Karlsruhe Institute of Technology (KIT) Karlsruhe Germany; ^3^ Graduate Program in Clean Technologies University of Cesumar Maringá Brazil

**Keywords:** biodiversity hotspots, conservation planning, habitat fragmentation, niche modeling

## Abstract

This study aimed to identify the number of vascular epiphytes endemic to the Amazon forest, estimate the distribution of 20 endemic vascular epiphytes (EVEs) using species distribution modeling (SDMs), and examine their potential occurrence inside and outside Amazonian Protected Areas (PAs) to predict potential habitats and conservation strategies. The study was carried out in the Amazon rainforest, focusing on PAs and their interactions with the distribution of EVEs. We used four primary sources of biodiversity data (GBIF, Kew Herbarium, Species Link, and Amazon inventories) to uncover the composition of endemic epiphytes. Fifteen EVEs were selected based on occurrence records from at least 17 locations. The research used a large dataset of epiphyte collections, eight modeling algorithms, and 34 environmental variables to generate consensus maps that identify suitable habitats for EVEs. The main results indicated that most EVEs have suitable areas concentrated along the edges of the Amazon rainforest, especially on the slopes of the Andes, with limited suitability in central and eastern Amazonia. The largest areas of suitability for the 20 model species were greater in unprotected areas (79%) than in protected areas (76.5%). The overlap between suitable areas and PAs highlights the importance of these areas in protecting EVEs. However, the significant presence of habitats outside PAs requires management strategies that go beyond their boundaries. The findings offer critical insights for biodiversity conservation and for planning actions to safeguard the diversity of Amazonian epiphytes in the face of increasing pressures.

## Graphical abstract

This study aimed to identify the number of vascular epiphytes endemic to the Amazon rainforest and to estimate the distribution of 15 of these species using species distribution modeling (SDM). In addition, the work examined the potential occurrence of these epiphytes inside and outside protected areas (PAs) in the Amazon, with a view to predicting suitable habitats and developing effective conservation strategies. The research was carried out in the Amazon rainforest, with an emphasis on the interactions between PAs and the distribution of endemic vascular epiphytes (EVEs). To achieve the objectives, four primary sources of biodiversity data, including GBIF and Kew Herbarium, were used to identify the composition of endemic epiphytes. Fifteen EVEs were selected based on records of occurrence in at least 16 locations. The investigation applied a large dataset of epiphyte collections, using eight modeling algorithms and 34 environmental variables to create consensus maps that identify suitable habitats for these species. The results showed that the majority of EVEs have suitable areas concentrated on the edges of the Amazon rainforest, especially on the slopes of the Andes, with less suitability in central and eastern Amazonia. The main conclusions emphasize the overlap between areas suitable for EVEs and PAs, highlighting the importance of these areas in protecting epiphytes. However, the significant presence of habitats outside PAs indicates the need for management strategies that also consider these regions, suggesting that conservation should go beyond the boundaries of protected areas.

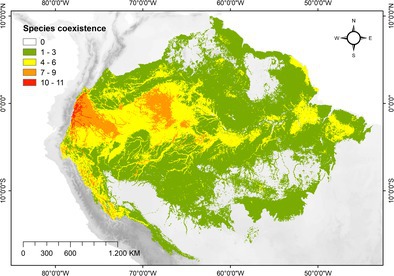

## 1 Introduction

The Amazon forest, one of the most biodiverse regions on Earth, is facing severe threats from habitat loss and fragmentation, deforestation, and climate change. These anthropogenic pressures have led to unprecedented shifts in species distribution, potentially leading to the extinction of up to 30% of animal and plant species within the next few decades, since estimates predict an increase of 1.5°C to 2.5°C in global temperature (Pereira et al. 2013). Such growing threats demand advanced analytical tools and technologies to enhance our understanding of species distributions and inform effective conservation strategies (Giannini et al. 2012).

Predictive modeling of species distribution has become an essential computational tool, combining species occurrence data with environmental variables (Anderson and Peterson 2003) to map current and potential distribution areas for both threatened and non‐threatened species (Alexandre et al. 2013). These models are valuable for predicting future suitable areas under different climate change scenarios (Giannini et al. 2012; Grüss et al. 2014; Virgili et al. 2017), assessing the effectiveness of Protected Areas (PAs) (Ferro et al. 2014), identifying new populations of endangered species (Kamino et al. 2012; Alexandre et al. 2013; Oliveira et al. 2016), and supporting restoration efforts to significantly improve success rates in the recovery of degraded or destroyed forest ecosystems (Amaral et al. 2021).

Protected areas are territorially delimited spaces with the main purpose of conserving and/or preserving related natural and cultural resources (Visconti et al. 2019). According to the International Union for Conservation of Nature (IUCN), these sites can be described as “terrestrial and/or marine areas designated especially for the protection and maintenance of biological diversity, as well as associated natural and cultural resources, and managed through legal instruments or other effective tools” (Dudley 2008). Globally, the establishment of PAs is a key strategy governments use to mitigate environmental degradation and conserve biodiversity (Visconti et al. 2019). These areas play a vital role in protecting species in situ, ensuring the continuity of ecosystem services and cultural values (MMA 2020). Additionally, PAs are crucial for the conservation of various biological groups, as they ensure the maintenance of ecological processes and the survival of a wide range of animal and plant species (Margules and Pressey 2000).

Many studies indicate that this vulnerability is accentuated by factors such as the inability to migrate in response to global warming and the edge and isolation effects in forest fragments. In addition, habitat restriction and low dispersal further increase the risk of extinction (Feeley and Silman 2011; Laurance et al. 2018; Lima et al. 2022). Therefore, epiphytes are essential components of forest biodiversity and can be useful bioindicators for monitoring environmental changes and habitat fragmentation. Their sensitivity to environmental changes makes them valuable indicators for studies of ecosystem health (Benzing 1990). They are important components for fauna and flora in general, providing food resources and microhabitats for fauna, as well as a reproductive refuge. The loss of epiphytes in tropical forests can lead to a cascading decline in the diversity of invertebrates and vertebrates that depend on them, affecting the entire food web (Nadkarni and Solano 2002). In addition, endemic epiphytes are often the first to disappear in disturbed landscapes, highlighting the necessary role of PAs for the resilience of this group (Zotz and Bader 2009; Gotsch et al. 2017).

In this context, the Amazon, a vast tropical forest, is crucial for conserving numerous animal and plant species, regulating the global climate, and sustaining critical ecological processes (Myers et al. 2000; Fearnside 2005; Cramer et al. 2004). In this way, epiphytes are a group that has been little studied, especially in the Amazon rainforest (Quaresma et al. 2022). These plants use a host plant (phorophyte) as support, developing either part of or all of their life cycle in the forest canopy (Quaresma et al. 2020). These plants take advantage of moisture and light in the tree canopy, forming microhabitats with resources and nutrients for various species of insects and birds (Zotz et al. 2021). They are very abundant in larger and older trees (Benzing 1990). Due to this dependence on an arboreal host, epiphytes are more vulnerable to climate change (Murakami et al. 2023), deforestation, temperature fluctuations, and land‐use changes (Zotz 2016).

Vascular epiphytes can be locally abundant and highly diverse, occurring from the understory to the periphery of tree canopies (Zotz and Hietz 2001). According to the latest estimates, the group currently comprises 31,311 species worldwide, distributed across 79 families, representing approximately 10% of the global vascular flora (Zotz et al. 2021). However, these plants are highly susceptible to environmental changes and deforestation, as they rely directly on other plants for survival (Murakami et al. 2023; Zotz et al. 2021). Endemic vascular epiphytes (EVEs) are species that are restricted to specific regions, and the Amazon, being a poorly studied forest, has a limited number of inventories and floristic collections (Quaresma et al. 2022; Lucas et al. 2025). This lack of data is particularly concerning, as this group is highly vulnerable to the impacts of climate change and land use (Myers et al. 2000; Klein et al. 2022).

Understanding the distribution of endemic plants is crucial for biodiversity conservation, especially in the Amazon, since these plants often play unique roles in ecosystems and can serve as important indicators of environmental health (Yilmaz et al. 2017). This knowledge also aids in making informed decisions about conservation strategies and the management of PAs. In this study, we utilized species distribution modeling (SDMs) and a large dataset of epiphyte collections (GBIF, Kew Herbarium, Species Link, and Amazon inventories) to (1) identify the number and identity of vascular epiphytes endemic to the Amazon; (2) model the potential distribution of selected endemic species; and (3) examine their potential occurrence inside and outside Amazonian PAs. Endemic vascular epiphytes (EVEs) in the Amazon rainforest face significant risks due to environmental changes, and the effectiveness of protected areas (PAs) in conserving these species depends on identification inside and outside PAs, highlighting areas suitable for the survival of EVEs. Our central hypothesis proposes that SDMs are an effective tool for implementing protective conservation measures for EVEs in the Amazon, as they can identify areas of high environmental suitability outside the current network of APs, with this delimitation being insufficient for the complete conservation of this group.

## 2 Materials and Methods

### 2.1 Study Area

We used the shapefile of the Amazon Forest and the PAs (Integrated Protection Areas, Sustainable Use Areas, and Indigenous Lands) from the Amazon Network of Georeferenced Socio‐Environmental Information (RAISG 2024) to delimit the study area. The Amazon is a tropical rainforest that extends from sea level to altitudes of over 3000 m in the Andes and Guiana Shield regions (Olson et al. 2001). Covering approximately 7 million km^2^, with more than half of the region located in Brazil, the region exhibits significant spatial and temporal rainfall heterogeneity, receiving between 2500 mm and 4000 mm of rainfall annually, making it the wettest region in the Americas (Fisch et al. 1998).

### 2.2 Occurrence Data

We used four primary biodiversity data sources for epiphyte assemblages: (i) the Global Biodiversity Information Facility (https://www.gbif.org/), (ii) the Kew Herbarium (https://powo.science.kew.org/), (iii) SpeciesLink (http://www.splink.org.br/), and (iv) epiphyte inventories carried out in the Amazon (Amazon Epiphytes Network). The taxonomic standardization and synonymy were verified using the Taxonomic Name Resolution Service (https://tnrs.biendata.org/), the checklist from Zotz et al. (2021), and World Flora Online (http://www.worldfloraonline.org/). The most recent synonym was selected when multiple names were suggested. Each occurrence point is derived from an observation, so all identifications have been validated (Crisp et al. 2001). The data were processed to eliminate duplicate records and records without geographical coordinates or confirmed species identity.

### 2.3 Species

**FIGURE 1:**
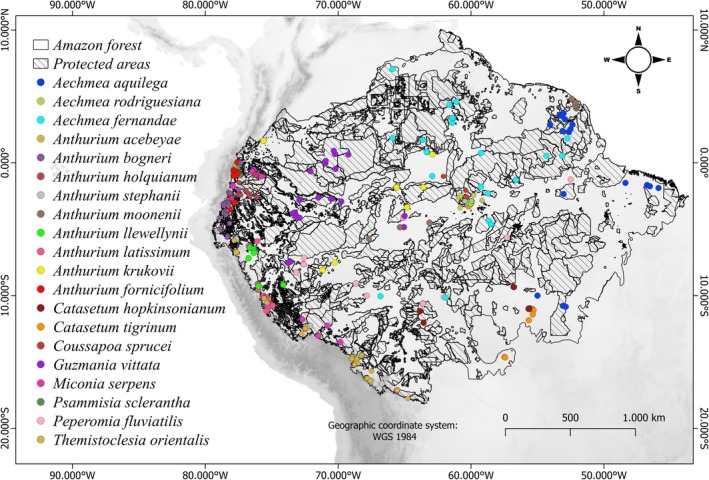
Distribution of the occurrence of the 20 EVE species. In light gray, the delimitation of the Amazon forest according to RAISG (2024).

To identify and determine Amazonian endemic species, we consulted the Reflora website (https://reflora.jbrj.gov.br), the Missouri Botanical Garden's Tropicos.org (https://tropicos.org/home), and World Flora Online (WFO) (https://www.worldfloraonline.org/). From there, we cataloged the richness of EVE with restricted occurrence in the Amazon basin (Annex 1; Supporting Information S1). However, for SDMs, we selected the 20 most abundant species, corresponding to seven families, using as a selection criterion their occurrence in at least 17 sites, necessary to meet the modeling prerequisites (Anderson and Peterson 2003; Wisz et al. 2008) (Figure 1; Annex 2—Supporting Information S2).

### 2.4 Environmental Data and Analysis

We utilized 34 environmental variables from various databases (Table 1) to model the distribution of EVEs at a resolution of 30 arcseconds (~1 km^2^). Environmental variables selected for modeling epiphyte distribution in the Amazon were chosen based on their documented ecological relevance for these organisms. Temperature and precipitation (CHELSA) are key drivers of epiphyte richness and distribution, influencing water availability, metabolic activity, and physiological limits (Wolf 1994; Zotz and Bader 2009; Kreft et al. 2004). Solar radiation and water vapor pressure (WorldClim 2.0) affect canopy microclimates by determining light availability and atmospheric humidity, both critical for photosynthesis and water balance in epiphytes (Wolf 1994; Gotsch et al. 2017). Forest, shrub, and pasture cover (FAO HWSD) serve as proxies for the structural habitat provided by host trees, with continuous forest cover supporting higher species richness and functional diversity (Küper et al. 2004). Elevation (SRTM) correlates with temperature, humidity, and cloud cover gradients, shaping altitudinal patterns of epiphyte diversity and endemism (Kessler 2002). Soil water stress (CGIAR) indirectly influences epiphytes through its effect on atmospheric moisture regimes and forest microclimates (Zotz and Hietz 2001; Murakami et al. 2023), while relative humidity (Climond) directly determines the capacity of epiphytes to intercept and absorb atmospheric water, a crucial factor in their survival and distribution (Benzing 1990; Hietz and Briones 1998). Together, these variables capture the main climatic, hydrological, and habitat structure gradients known to influence epiphyte occurrence in tropical forests.

**TABLE 1:** List of variables used and their respective databases and URLs.

Dataset	URL	Variables
Temperature and Precipitation (19 variables)	CHELSA (http://chelsa‐climate.org/)	BIO1 = Annual Mean Temperature BIO2 = Mean Diurnal Range (Mean of monthly (max temp—min temp)) BIO3 = Isothermality (BIO2/BIO7) (×100) BIO4 = Temperature Seasonality (standard deviation ×100) BIO5 = Max Temperature of Warmest Month BIO6 = Min Temperature of Coldest Month BIO7 = Temperature Annual Range (BIO5‐BIO6) BIO8 = Mean Temperature of Wettest Quarter BIO9 = Mean Temperature of Driest Quarter BIO10 = Mean Temperature of Warmest Quarter BIO11 = Mean Temperature of Coldest Quarter BIO12 = Annual Precipitation BIO13 = Precipitation of Wettest Month BIO14 = Precipitation of Driest Month BIO15 = Precipitation Seasonality (Coefficient of Variation) BIO16 = Precipitation of Wettest Quarter BIO17 = Precipitation of Driest Quarter BIO18 = Precipitation of Warmest Quarter BIO19 = Precipitation of Coldest Quarter
Solar radiation (3 variables) and Water vapor pressure (3 variables)	WorldClim 2.0 (http://worldclim.org/version2)	SolarRadiationMin SolarRadiationMean SolarRadiationMax Mean MODIS cloud cover (Cld) Distance to oceanic coast (cdist) Elevation (elev)
Forest, shrub, and pasture cover (1 variable)	http://www.fao.org/soils‐portal/soil‐survey/soil‐maps‐and‐databases/harmonized‐world‐soil‐database‐v12/en/	EnhacendVegetation
Elevation (1 variable)	NASA Shuttle Radar Topographic Mission (SRTM) (http://srtm.csi.cgiar.org/)	Elev 5 km (EVIrng5km)
Soil Water Stress (1 variable)	Global High‐Resolution Soil‐Water Balance (http://www.cgiar‐csi.org/data/global‐high‐resolution‐soil‐water‐balance#download)	Water table depth (WTD)
Relative humidity (6 variables)	Climond (https://www.climond.org/RawClimateData.aspx)	Relative humidity 9amMin Relative humidity 9amMean Relative humidity 9amMax Relative humidity 3pmMin Relative humidity 3pmMean Relative humidity 3pmMax

To avoid overfitting, we applied the variance inflation factor (VIF) to test multicollinearity among variables in R 4.2.2 (R Core Team 2020), retaining 11 uncorrelated predictors (VIF ≤ 3.0, correlation < 0.85). The species distribution models (SDMs) were developed using eight algorithms available in the BIOMOD2 package (Thuiller et al. 2019): (1) a regression method (generalized linear models—GLM; McCullagh and Nelder 1989); (2) four machine learning methods or complex methods (artificial neural networks—ANN; Hopfield 1982), generalized boosted models—GBM (Friedman et al. 2000), Maximum Entropy—MaxEnt (Phillips et al. 2006), and random forests—RF (Breiman 2001); (3) two classification methods (CTA Tree Analysis – ANN; Hopfield 1982), flexible discriminant analysis—FDA (Hastie et al. 1994); (4) Surface Envelope—SRE model, similar to Bioclim (Jiguet et al. 2010). The script is available at the link (https://github.com/pedroeisenlohr/niche_modelling).

As the database used for the study only contains records of species presence, pseudo‐absence points were generated randomly to adjust the models. To assess the model's accuracy and adjust it, the occurrence data were divided into training and test sets at a ratio of 30% and 70%, respectively. This procedure was repeated 10 times to enhance model robustness. The predictive power of the individual algorithms was tested using the TSS (*True Skill Statistic*), excluding algorithms with TSS values < 0.4 from the final consensus models (Lopes et al. 2023). The final models were based on algorithms with TSS ≥ 0.4, which were combined to generate consensus maps showing areas with high species occurrence probability.

We quantified model uncertainty using the average receiver operating characteristic (ROC), standard deviation (SD), and uncertainty coefficient, with sensitivity values (i.e., true positive rate) used as a reliability measure (Thuiller et al. 2019). Models were ranked based on sensitivity and SD as follows: poor (sensitivity ≤ 50 or SD ≥ 50), average (sensitivity ≥ 50 with SD ≤ 45), good (sensitivity ≥ 70 with SD ≤ 30), and optimal (sensitivity ≥ 90 with SD ≤ 30) (Lopes et al. 2023).

Potential distribution maps for the Amazon were created in ArcGIS 10.8 software. Binary suitability/non‐suitability maps were generated using the Reclassify tool in Spatial Analyst with a threshold of 0.5, corresponding to a conservative threshold representing areas with moderate to high suitability probabilities, ensuring greater accuracy in the results (Bertelsmeier and Courchamp 2014). We compared the size and suitable area changes in EVE's areas inside and outside PAs (RAISG 2024). The size of the suitable area (SA) in the consensus models was calculated by the difference between the number of pixels in each class (0 = not suitable; 1 = suitable). The equation used (Lopes et al. 2023) was: $\mathrm{SA}\%=\left(\frac{\;N^{{}^{\circ}}\;\text{of pixels in the appropriate area for the species}}{\;N^{{}^{\circ}}\;\text{pixel totals}}\right)\times 100.$

## 3 Results

**FIGURE 2:**
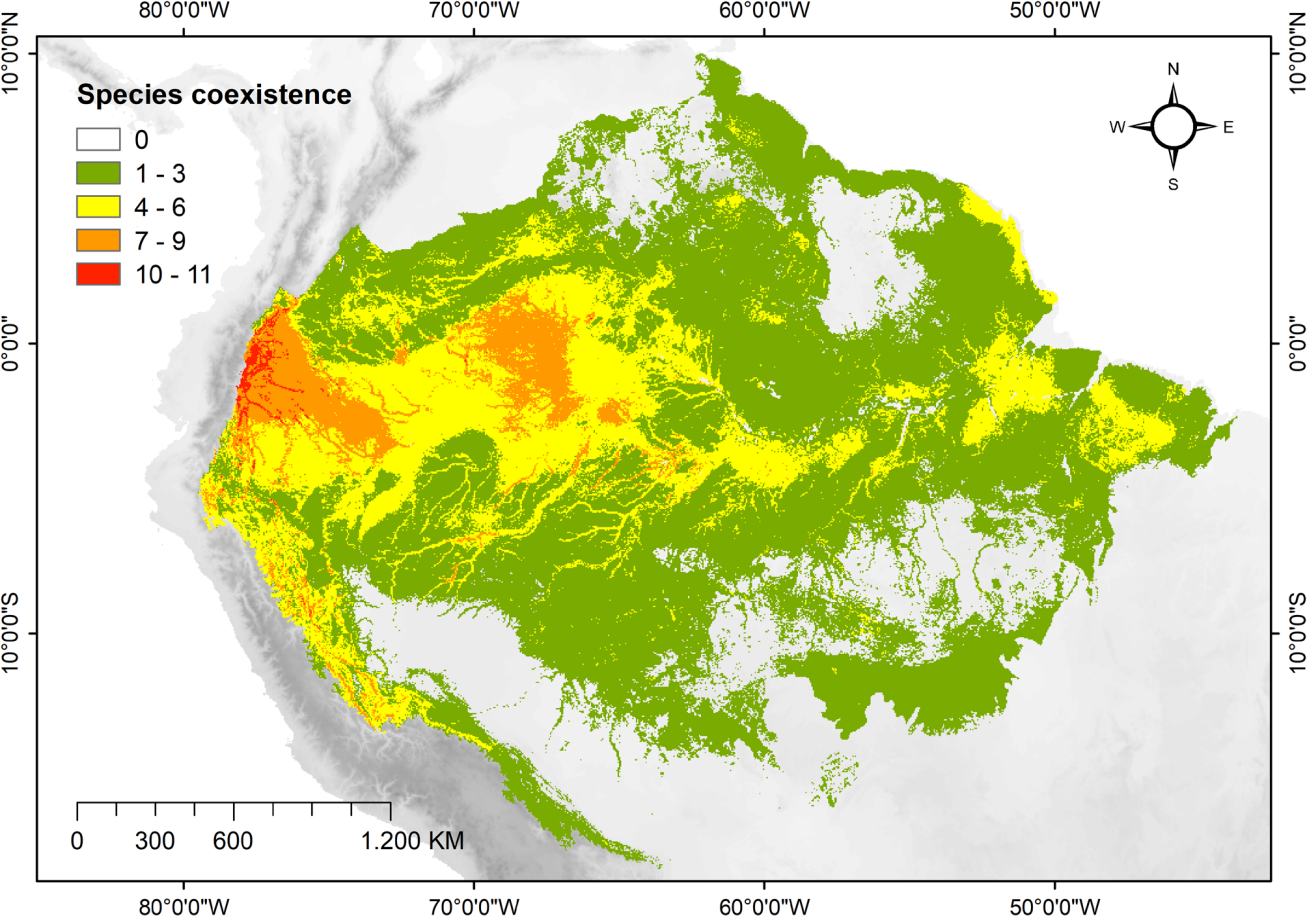
Current potential distribution of endemic vascular epiphytes (EVEs) in the Amazon forest. Suitability indicates the co‐occurrence of species in their potential distribution.

A total of 178 species endemic to the Amazon were cataloged. The genera with the highest number of endemic species were *Anthurium* (38 spp), *Lepanthes* (24 spp), and *Catasetum* (13 spp). Ninety‐two species classified as endemic belong to the Orchidaceae family (52%) (Annex 1; Supporting Information S1). According to the final consensus models of species distribution, the majority of endemic species revealed areas of suitability along the Amazon's edges, particularly at altitudes between 2000 and 3000 m on the western Andean slope (Figure 2). These regions coincide with relevant PAs, whereas central, northern, southern, and eastern Amazonia exhibited low suitability for endemic vascular epiphytes (EVEs).

The selected predictor variables for the models, based on VIF (≤ 3.0), included bio 02, bio 03, bio 08, bio 15, bio 18, bio 19, elevation, humidity (9amMax), solar radiation, and water table depth (WTD). The mean ROC sensitivity values (± standard deviation) ranged from 83.74 (±13.14) to 97.27 (±4.20), indicating good reliability for most models. The performance of the algorithms was satisfactory, with 60% of the final consensus models (12 spp.) represented by “excellent” reliability and robustness, 25% (five spp.) with “good” reliability results, 10% (two spp.) with “medium” reliability, and only 5% (one spp.) with “low” reliability. The SRE algorithm was excluded from all consensus models due to a TSS value below 0.4, and the GBM algorithm was discarded for most models due to inadequate performance (Annex 3). Overall, the suitable areas for the species were low, reflecting the restrictive distribution of these endemic species in the biogeographic context. The species with the highest areas of suitability in the Amazon Forest were *Coussapoa sprucei* (63%), *Aechmea rodriguesiana* (53%), and *Anthurium bogneri* (21%). In contrast, the other species had suitability indices below 2%. The maximum number of overlapping species, ten to eleven, occurs on the western edge of the Amazon forest.

**FIGURE 3:**
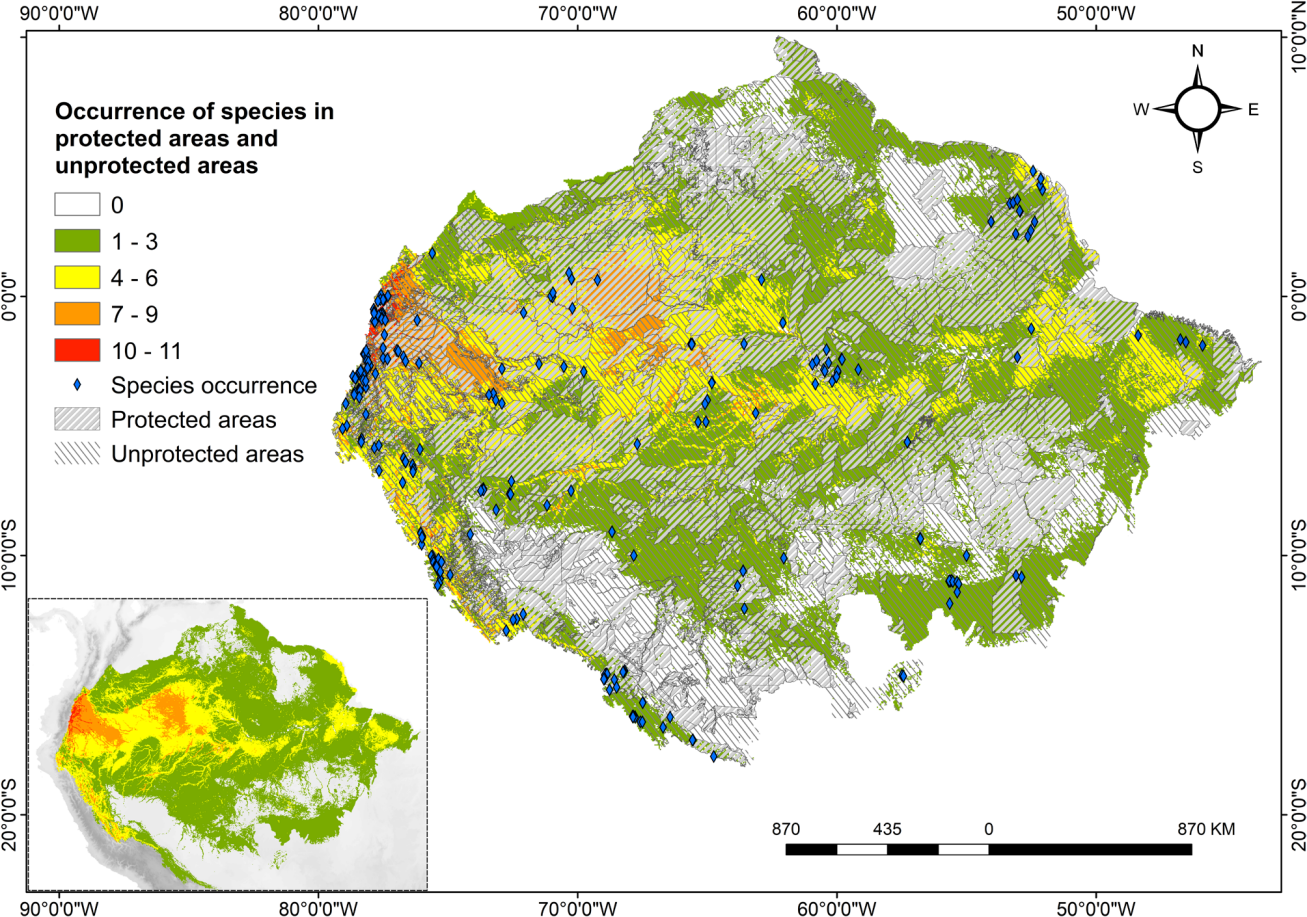
Potential distribution of EVEs occurring together within protected areas and in unprotected areas.

When we analyzed suitability inside and outside protected areas (Figure 3), the highest suitability values were found for the coexistence of seven to eleven species on the western slope of the Andes. The species *C. sprucei* showed the highest suitability (49%), followed by *A. rodriguesiana* (39%), *A. bogneri* (24%), and *Aechmea fernandae* (19%). For unprotected areas, a similar pattern of species occurrence was observed on the western slopes of the Andes in the Amazon, with some species occurring more frequently outside than inside the boundaries of protected areas ( *C. sprucei* , 62%, and *A. rodriguesiana*, 54%). However, the largest areas of suitability for the 20 model species were greater in unprotected areas (79%) than in protected areas (76.5%) (Table 2).

**TABLE 2:** Values of the suitable area of occurrence inside and outside the Amazonian PAs for the 20 EVEs analyzed.

Species	Suitable total area		
	Protected areas	Unprotected areas	Amazon forest
*Coussapoa sprucei* Mildbr.	49%	63%	62%
*Aechmea rodriguesiana* (L.B. Sm.) L.B. Sm.	39%	54%	53%
*Anthurium bogneri* Croat	24%	16%	21%
*Aechmea fernandae* (E. Morren) Baker	19%	19%	20%
*Guzmania vittata* (Mart. ex Schult. f.) Mez	17%	14%	17%
*Aechmea aquilega* (Salisb.) Griseb.	11%	14%	17%
*Anthurium holquianum* Croat & D.C.Bay	12%	6%	9%
*Peperomia fluviatilis* Yunck.	5%	9%	7%
*Anthurium krukovii* Croat	7%	6%	7%
*Anthurium latissimum* Engl.	3%	4%	5%
*Miconia serpens* (Triana) Cogn.	5%	2%	4%
*Anthurium acebeyae* Croat	3%	3%	3%
*Anthurium moonenii* Croat & E.G.Gonç.	3%	5%	3%
*Anthurium stephanii* Croat & Acebey	3%	3%	3%
*Anthurium fornicifolium* Croat	2%	1%	1%
*Themistoclesia orientalis* Luteyn	1%	2%	1%
*Catasetum hopkinsonianum* G.F. Carr & V.P. Castro	1%	1%	1.2%
*Catasetum tigrinum* Rchb. f.	0.5%	2%	1%
*Anthurium llewellynii* Croat	0.4%	1%	1.0%
*Psammisia sclerantha* A.C. Sm.	0.3%	0.2%	0.3%
Total	76.5%	79%	78.5%

## 4 Discussion

The results of this study indicate a very clear and well‐known distribution in the literature for epiphytes, including the coexistence of endemic vascular epiphytes (EVEs) in the Amazon. Furthermore, this distribution is significantly influenced by climatic and topographic variables, particularly at altitudes between 2000 and 3000 m on the western slopes of the Andes, regions that contain the areas of greatest suitability for the modeled EVEs. However, this discovery exposes a dangerous flaw in the current Amazon conservation strategy. If climatically suitable habitats for these endemic and specialized species are predominantly located outside protected areas and precisely at the most threatened forest edges, then our current conservation model is not only insufficient but potentially flawed. We are effectively protecting the core of the ecosystem while leaving its most unique and vulnerable species exposed on the periphery. These findings underscore the need for targeted management and conservation strategies in the most suitable areas identified, particularly those supporting multiple endemic species, to ensure the protection and persistence of these vulnerable populations.

The distribution patterns of vascular epiphytes in the Amazon, especially their concentration in specific regions such as the western slopes of the Andes, can be explained by the biological and ecological characteristics of these plants (Zotz and Bader 2009). However, endemic species are generally associated with particular microclimates, making them much more sensitive to changes in temperature and humidity (Gentry and Dodson 1987; Krömer et al. 2005). Therefore, small changes in these factors may act to different degrees compared to more generalist species, leading to large population declines in endemic species (Murakami et al. 2023). At altitudes between 2000 and 3000 m, some highland specialist species, such as those found on the western slopes of the Andes, may benefit from a combination of environmental factors: high humidity, frequent cloud cover, and moderate temperatures, which promote efficient absorption of water directly from the atmosphere and enhance photosynthesis (Nadkarni and Solano 2002; Taylor et al. 2022). These conditions are less common at lower elevations or in regions with more extreme climates, such as central, northern, southern, and eastern Amazonia, where events such as extreme dry seasons and higher temperatures can limit the colonization and survival of generalist lowland epiphytes (Benzing 1990; Zotz and Bader 2009; Schongart et al. 2024). When focusing on high‐altitude species, epiphytes are known to depend on phorophytes with specific characteristics, such as height, canopy structure, and mosses or lichens, further restricting their distribution to specific habitats (Gradstein et al. 2003). In studies with interaction networks, epiphytes demonstrate colonization preferences; for example, taller trees with larger diameters and rougher bark facilitate attachment and tend to host more epiphytes compared to hosts of lower height, smaller diameters, and smooth bark (Woods et al. 2015; Ceballos et al. 2016). This ecological requirement, however, makes them particularly vulnerable, as recent evidence suggests that older, larger trees may be dying at accelerated rates due to climate change (Aleixo et al. 2019), which would have a direct and negative impact on the survival of epiphyte populations. However, comprehensive data on the distribution of phorophytes in the Amazon are still scarce, particularly on the colonization patterns of epiphytic host trees at altitude (Pacheco and Barberena 2021). Our results reveal a critical conservation gap, with the vast majority (79%) of suitable habitats for EVEs located outside the Amazonian Protected Areas (PAs) network. This spatial mismatch between high‐suitability habitats and the large number of conservation areas exposes these specialized species to significant threats, especially considering that the most suitable areas are concentrated along rapidly transforming forest edges, such as the Andean slopes. This pattern aligns with the growing body of evidence on the “conservation paradox” in the tropics, where PAs are often established in remote, low‐conflict areas (except Indigenous territories), inadvertently leaving regions of high ecological value and vulnerability unprotected (Jenkins and Joppa 2009; Venter et al. 2018).

The concentration of EVEs in unprotected zones suggests that the current PA system, while crucial for safeguarding core forest habitats, is insufficient for the conservation of this key ecological group. This requires a shift from an exclusive focus on strengthening existing PAs to a landscape‐scale approach that integrates biodiversity conservation strategies in human‐modified matrices (Melo et al. 2013). Overall, the potential occurrence of EVEs was considerably limited across much of the forest, with few species having a large area of suitability and many species having little suitable area. Studies using niche modeling have concluded with similar results: in the tropical forests of Java for the endemic epiphyte *Crepidium ridleyi* (J.J.Sm.) Szlach. (Usmandi et al. 2023) and other plant groups, such as endemic species of Erythroxylaceae in Brazil (Cordeiro 2013), and baobab tree species (*Adansonia* spp.) (Malvaceae) endemic to the forests of Madagascar (Wan et al. 2021).

Endemic species often experience long periods of geographic isolation, resulting in highly specialized adaptations that make them dependent on local ecological conditions. Consequently, their restricted gene pool drastically limits their adaptive response to habitat changes, whether resulting from land‐use changes, pollution, the introduction of invasive species, or the effects of climate change (Yessoufou et al. 2012; Yilmaz et al. 2017). Given this remarkable susceptibility, conserving endemic biodiversity requires an approach that transcends the boundaries of traditional conservation units. The creation of protected areas, while essential, is insufficient on its own, as the results of this study show. This need becomes absolutely critical in regions suffering from intense degradation processes, such as the eastern and southern Amazon, where alarming rates of deforestation have already catalyzed severe and possibly irreversible population declines in the unique fauna and flora of these locations (Ohana et al. 2015; Middleton 2016).

Given this alarming scenario, our projections emphasize that species inhabiting higher elevations may be less affected by climate change, and that geographic isolation in the mountains acts as a physical barrier that leads to the emergence of refugia, i.e., microhabitats favorable to the establishment of some species compared to the eastern part of the Amazon (Zhu et al. 2017). Western Amazonia was one of the least affected by climate change during the Pleistocene (Kreft et al. 2004), a factor not observed in the lower plains, where the potential climate impact on wetlands is a significant concern for functionality and biodiversity (Mano et al. 2023).

In general, the results demonstrated that suitable areas for EVEs extend beyond the boundaries of protected areas, emphasizing the need for targeted conservation actions in unprotected regions. Ecological restoration strategies should prioritize regions of high suitability, particularly in the Western Amazon, where species overlap is most significant. Public policies can integrate these findings by promoting land‐use mitigation practices in priority regions, implementing ecological corridor strategies, thereby increasing habitat connectivity and supporting the dispersal of endemic epiphytes. Furthermore, designating new protected areas in critical zones, such as the Andean slopes, could effectively safeguard local biodiversity. These actions are aligned with global biodiversity conservation goals, such as those outlined in the post‐2020 Global Biodiversity Framework (Visconti et al. 2019).

## 5 Conclusions

The results corroborate the initial hypothesis, revealing a critical conservation gap: the majority (79%) of suitable habitat for EVEs lies outside the protected area system. This gap is particularly alarming, as the zones most suitable for models, such as the Andean slopes. Therefore, this study demonstrates the urgency of moving beyond the current ‘islands of protection’ strategy. Conservation policies must integrate species distribution modeling to actively prioritize the protection of these unprotected key areas, thus ensuring the resilience of these species and the Amazon ecosystem to climate change.

## Author Contributions

**Keller Maurício dos Santos Lucas:** conceptualization (lead), data curation (lead), methodology (lead), visualization (lead), writing – original draft (lead), writing – review and editing (equal). **Adriano Quaresma:** conceptualization (supporting), formal analysis (supporting), investigation (supporting), methodology (supporting), supervision (equal), writing – review and editing (supporting). **Giuliette Barbosa Mano:** conceptualization (supporting), methodology (supporting), visualization (supporting), writing – review and editing (supporting). **Maria Teresa Fernandez Piedade:** conceptualization (supporting), supervision (supporting), writing – review and editing (supporting). **Viviane Pagnussat Klein:** conceptualization (supporting), supervision (supporting), writing – review and editing (supporting). **Layon Oreste Demarchi:** conceptualization (supporting), supervision (supporting), writing – review and editing (supporting). **Jochen Schöngart:** conceptualization (supporting), supervision (supporting), writing – review and editing (supporting). **Aline Lopes:** conceptualization (supporting), formal analysis (supporting), investigation (supporting), methodology (supporting), visualization (supporting), writing – review and editing (supporting).

## Disclosure

*Animal Research*: This study has not involved animal experiments.

## Conflicts of Interest

The authors declare no conflicts of interest.

## Supporting information


**Annex 1.** Supporting Information.

**Annex 2.** Supporting Information.

**Annex 3.** Supporting Information.

**Appendix S1:** Supporting Information.

**Appendix S2:** Supporting Information.

## Data Availability

I declare that all supplementary data related to this article is included in the main file of my submission. Furthermore, I affirm that access to all necessary data files will be provided to the editors and reviewers for evaluation and revision purposes.
