# Supplementary material for: Endemic Vascular Epiphytes: Integrating Protected Areas and Suitability Models in the Amazon Forest
Source: Ecol Evol. 2025 Nov 29;15(12):e72407. doi: 10.1002/ece3.72407 (PMC12663693; doi:10.1002/ece3.72407)
Supplement: Supplementary file 1 — Annex 1. Supporting Information. [file ECE3-15-e72407-s001.docx]

**Annex 1.** List of endemic vascular epiphyte species to the Amazon Biome.

| **Families** | **Species** | **Nº records** |
| --- | --- | --- |
| **Araceae** | *Anthurium acebeyae* Croat | 47 |
|  | *Anthurium alatum* Engl*.* | 2 |
|  | *Anthurium apanui* Croat | 2 |
|  | *Anthurium bogneri* Croat | 55 |
|  | *Anthurium chinchipense* Croat & Lingán | 4 |
|  | *Anthurium chocoense* Croat | 12 |
|  | *Anthurium cremersii* G*.*S.Buntinge x Croat | 12 |
|  | *Anthurium debilipeltatum* Croat | 6 |
|  | *Anthurium fornicifolium* Croat | 17 |
|  | *Anthurium gehrigeri* Croat | 1 |
|  | *Anthurium hamiltonii* Croat & Lingán | 2 |
|  | *Anthurium holquinianum* Croat & D.C.Bay | 18 |
|  | *Anthurium huallagense* Engl*.* | 9 |
|  | *Anthurium huashikatii* Croat | 2 |
|  | *Anthurium icanense* G*.*M*.* Barroso | 1 |
|  | *Anthurium iramireziae* G*.*S*.*Bunting | 1 |
|  | *Anthurium josei* Croat | 1 |
|  | *Anthurium kayapii* Croat | 8 |
|  | *Anthurium knappiae* Croat | 3 |
|  | *Anthurium krukovii* Croat | 20 |
|  | *Anthurium latissimum* Engl*.* | 28 |
|  | *Anthurium llewellynii* Croat | 18 |
|  | *Anthurium magdae* Croat & Lingán | 5 |
|  | *Anthurium miaziense* Croat | 5 |
|  | *Anthurium moonenii* Croat & E.G.Gonç. | 18 |
|  | *Anthurium mostaceroi* Croat | 2 |
|  | *Anthurium oxyanthum* Croat & D.C.Bay | 1 |
|  | *Anthurium oxyphyllum* Sodiro | 3 |
|  | *Anthurium palacioanum* Croat | 8 |
|  | *Anthurium palmarense* Croat | 1 |
|  | *Anthurium pradoense* Croat | 3 |
|  | *Anthurium quipuscoae* Croat | 2 |
|  | *Anthurium stephanii* Croat & Acebey | 28 |
|  | *Anthurium tarapotense* Engl*.* | 9 |
|  | *Anthurium timplowmanii Croat* | 3 |
|  | *Anthurium vaupesianum* Croat | 15 |
|  | *Anthurium warintsense* Croat | 1 |
|  | *Anthurium yarumalense* Engl*.* | 1 |
| **Araliaceae** | *Sciodaphyllum lancifoliolatum* (Frodin) Lowry, G.M. Plunkett & M.M. Mora | 2 |
| **Bromeliaceae** | *Aechmea aquilega* (Salisb.) Griseb. | 30 |
|  | *Aechmea egleriana* L.B. Sm. | 9 |
|  | *Aechmea fernandae* (E. Morren) Baker | 43 |
|  | *Aechmea murcae* (L.B. Sm.) L.B. Sm. & M.A. Spencer | 1 |
|  | *Aechmea polyantha* E. Pereira & Reitz | 3 |
|  | *Aechmea prancei* L.B. Sm. | 1 |
|  | *Aechmea rodriguesiana* (L.B. Sm.) L.B. Sm. | 27 |
|  | *Guzmania alcantareoides* H. Luther | 1 |
|  | *Guzmania cuatrecasasii* L.B. Sm. | 1 |
|  | *Guzmania goudotiana* Mez | 1 |
|  | *Guzmania pungens* L.B. Sm. | 1 |
|  | *Guzmania triangularis* L.B. Sm. | 1 |
|  | *Guzmania vittata* (Mart. ex Schult. f.) Mez | 40 |
|  | *Racinaea euryelytra* J.R. Grant | 5 |
|  | *Tillandsia membranacifolia* L.B.Sm. | 2 |
| **Clusiaceae** | *Clusia guaviarensis* Cuatrec*.* | 9 |
| **Dryopteridaceae** | *Elaphoglossum bryogenes* Mickel | 3 |
| **Ericaceae** | *Cavendishia speciosa* A.C. Sm. | 1 |
|  | *Ceratostema pedunculatum* Luteyn | 2 |
|  | *Ceratostema silvícola* A.C. Sm. | 5 |
|  | *Diogenesia antioquiensis* Luteyn | 1 |
|  | *Macleania recumbens* A.C. Sm. | 1 |
|  | *Orthaea oriens* Luteyn | 7 |
|  | *Psammisia sclerantha* A.C. Sm. | 55 |
|  | *Themistoclesia orientalis* Luteyn | 22 |
|  | *Thibaudia lateriflora* A.C. Sm. | 5 |
|  | *Thibaudia yungensis*Luteyn & Pedraza | 2 |
| **Gesneriaceae** | *Columnea chrysotricha* L.E. Skog & L.P. Kvist | 17 |
|  | *Columnea coronocrypta* M. Amaya, L.E. Skog & L.P. Kvist | 1 |
|  | *Columnea cuspidata* L.E. Skog & L.P. Kvist | 1 |
|  | *Drymonia laciniosa* Wiehler | 1 |
| **Hymenophyllaceae** | *Hymenophyllum prionema* Kunzeex J.W. Sturm | 1 |
| **Marcgraviaceae** | *Marcgraviastrum apaporensis* de Roon & Bedell | 2 |
| **Melastomataceae** | *Blakea andreana* Cogn. | 2 |
|  | *Blakea calyptrata* Gleason | 7 |
|  | *Blakea granatensis* Naudin | 1 |
|  | *Blakea truncata* Gleason | 7 |
|  | *Miconia serpens* (Triana) Cogn. | 54 |
| **Moraceae** | *Ficus tequendamae* Dugand | 1 |
| **Orchidaceae** | *Anathallis taracuana* (Schltr.) F. Barros & L.R.S. Guim. | 2 |
|  | *Campylocentrum tenue* (Lindl.) Rolfe | 1 |
|  | *Catasetum albovirens*Barb. Rodr. | 4 |
|  | *Catasetum ariquemense* F.E.L. Miranda & K.G. Lacerda | 1 |
|  | *Catasetum complanatum* F.E.L. Miranda & K.G. Lacerda | 3 |
|  | *Catasetum denticulatum* Miranda | 5 |
|  | *Catasetum galeritum* Rchb. f. | 7 |
|  | *Catasetum gnomus* Linden & Rchb. f. | 7 |
|  | *Catasetum hopkinsonianum* G.F. Carr & V.P. Castro | 22 |
|  | *Catasetum kraenzlinianum*Mansf. | 9 |
|  | *Catasetum multifidum* F.E.L. Miranda | 10 |
|  | *Catasetum rivularium* Barb. Rodr. | 4 |
|  | *Catasetum rondonense* Pabst | 2 |
|  | *Catasetum semicirculatum* F.E.L. Miranda | 6 |
|  | *Catasetum tigrinum* Rchb. f. | 32 |
|  | *Cattleya trianae* Linden & Rchb. f. | 1 |
|  | *Dichaea caquetana* Schltr. | 1 |
|  | *Dracula dalessandroi* Luer | 1 |
|  | *Dracula papillosa* Luer & Dodson | 1 |
|  | *Dracula rezekiana* Luer & R. Hawley | 3 |
|  | *Dracula vlad-tepes*Luer & R.Escobar | 1 |
|  | *Dryadella osmariniana* (Braga) Garay & Dunst. | 2 |
|  | *Elleanthus auriculatus* Garay | 2 |
|  | *Elleanthus ecuadorensis* Garay | 9 |
|  | *Elleanthus lateralis* Garay | 4 |
|  | *Elleanthus phorcophyllus* Garay | 1 |
|  | *Elleanthus rhizomatosus* Garay | 2 |
|  | *Eloyella dalstroemii* Dodson | 1 |
|  | *Encyclia yauaperyensis* (Barb.Rodr.) Porto & Brade | 1 |
|  | *Epidendrum acreense* (Brieger& Bicalho) Christenson | 1 |
|  | *Epidendrum arachnoglossum* Rchb.f. ex André | 1 |
|  | *Epidendrum jatunsachanum* Dodson & Hágsater | 1 |
|  | *Epidendrum lezlieae* R. Vásquez & Ibisch | 1 |
|  | *Epidendrum porquerense* F. Lehm. & Kraenzl. | 1 |
|  | *Epidendrum stenostachyum* Hágsater & E. Santiago | 14 |
|  | *Fernandezia stuebelii* (Schltr.) M.W. Chase | 12 |
|  | *Gongora charontis* Rchb. f. | 1 |
|  | *Ionopsis zebrina* Kraenzl. | 1 |
|  | *Kefersteinia koechliniorum* Christenson | 3 |
|  | *Laelia schultzei* (Schltr.) J.M.H. Shaw | 1 |
|  | *Lepanthes allector* Luer | 1 |
|  | *Lepanthes alopex* Luer & Hirtz | 3 |
|  | *Lepanthes asoma* Luer & Hirtz | 1 |
|  | *Lepanthes calliope* Luer & Hirtz | 1 |
|  | *Lepanthes caloura* Luer & Hirtz | 3 |
|  | *Lepanthes calypso* Luer & Hirtz | 1 |
|  | *Lepanthes conjuncta* Luer & Hirtz | 2 |
|  | *Lepanthes dalessandroi* Luer | 2 |
|  | *Lepanthes deleastes* Luer | 13 |
|  | *Lepanthes echo* Luer & Hirtz | 1 |
|  | *Lepanthes eumeces* Luer | 1 |
|  | *Lepanthes flexuosa* Luer | 1 |
|  | *Lepanthes gaileana* Luer & Hirtz | 2 |
|  | *Lepanthes homotaxis*Luer | 6 |
|  | *Lepanthes illex* Luer | 1 |
|  | *Lepanthes inamoena* Luer | 4 |
|  | *Lepanthes iricolor* Luer & Hirtz | 1 |
|  | *Lepanthes lloensis* Luer | 1 |
|  | *Lepanthes nontecta* Luer | 2 |
|  | *Lepanthes ortegae* Luer & Hirtz | 1 |
|  | *Lepanthes polytricha* Luer | 1 |
|  | *Lepanthes pubescens* Luer | 1 |
|  | *Lepanthes schizura* Luer | 3 |
|  | *Lepanthes ximenae* Luer | 4 |
|  | *Lepanthopsis ubangii* Luer | 1 |
|  | *Macroclinium brasiliense* (Pabst) Dodson | 1 |
|  | *Masdevallia heteroptera* Rchb. f. | 2 |
|  | *Masdevallia panguiensis* Luer & Andreetta | 2 |
|  | *Masdevallia venatoria* Luer & Malo | 1 |
|  | *Maxillaria camaridioides* Schltr. | 1 |
|  | *Maxillaria dalessandroi* Dodson | 4 |
|  | *Maxillaria melina* Lindl. | 4 |
|  | *Maxillaria schultzei* Schltr. | 1 |
|  | *Maxillaria thurstoniorum* Dodson | 6 |
|  | *Maxillaria tocotana* Schltr. | 9 |
|  | *Maxillaria whittenii* Dodson | 7 |
|  | *Mormodes elegans* Miranda | 3 |
|  | *Mormodes paraensis* Salazar & J.B.F. Silva | 13 |
|  | *Notylia yauaperyensis* Barb. Rodr. | 7 |
|  | *Oncidium praenitens* (Rchb. f.) M.W. Chase & N.H. Williams | 1 |
|  | *Oncidium trachycaulon* Schltr. | 6 |
|  | *Ornithocephalus suarezii* Dodson | 5 |
|  | *Paphinia herrerae* Dodson | 3 |
|  | *Platystele consobrina* Luer | 1 |
|  | *Platystele edmundoi* Pabst | 11 |
|  | *Platystele lawessonii* Luer | 2 |
|  | *Platystele viridis* Luer | 2 |
|  | *Plectrophora calcarhamata* Hoehne | 3 |
|  | *Pleurothallis brachiata* Luer | 8 |
|  | *Pleurothallis lemniscifolia* Luer | 1 |
|  | *Pleurothallis sphaerantha* Luer | 10 |
|  | *Porroglossum condylosepalum* Sweet | 1 |
|  | *Rodriguezia carnea* Lindl. | 1 |
|  | *Stelis bigibba* Schltr. | 1 |
| **Piperaceae** | *Peperomia acreana* C. DC. | 4 |
|  | *Peperomia blackii* Yunck. | 1 |
|  | *Peperomia fluviatilis* Yunck. | 25 |
| **Urticaceae** | *Coussapoa arachnoidea* Akkermans & C.C. Berg | 2 |
|  | *Coussapoa cinnamomea* Cuatrec. | 3 |
|  | *Coussapoa sprucei* Mildbr. | 17 |
| **Total** |  | 1159 |

Based on consultations of the sites described in Materials and methods.
