# Supplementary material for: Endemic Vascular Epiphytes: Integrating Protected Areas and Suitability Models in the Amazon Forest
Source: Ecol Evol. 2025 Nov 29;15(12):e72407. doi: 10.1002/ece3.72407 (PMC12663693; doi:10.1002/ece3.72407)
Supplement: Supplementary file 2 — Annex 2. Supporting Information. [file ECE3-15-e72407-s005.docx]

**Annex 2.** We selected 20 species based on the number of occurrence points. A number below 17 would hinder the final modeling results. Therefore, we standardized the occurrence points of the modeled species above 17.

| **Families** | **Species** | **Nº records** | **Occurrence** |
| --- | --- | --- | --- |
| **Araceae** | *Anthurium acebeyae* Croat | **47** | **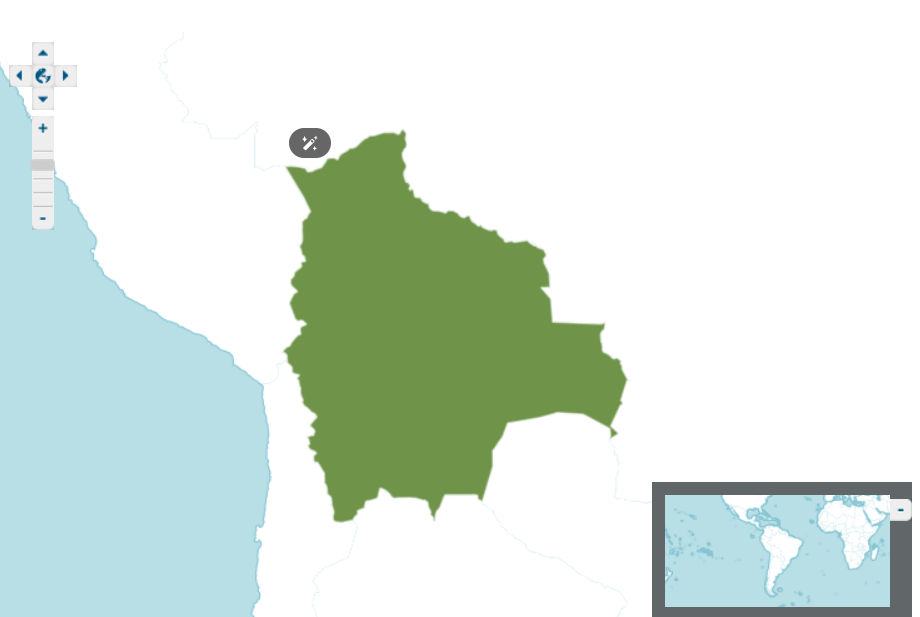** |
|  | *Anthurium bogneri* Croat | **55** | **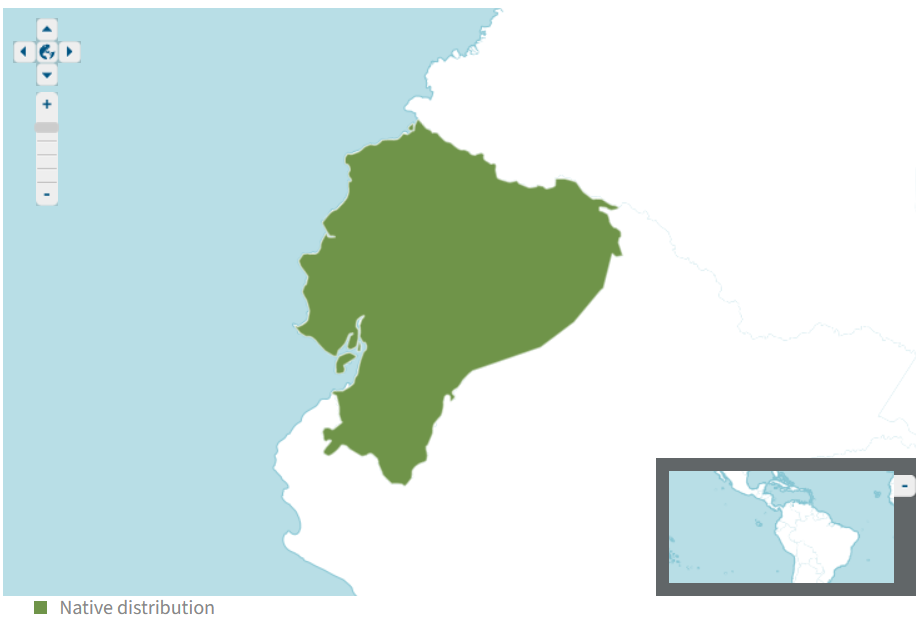** |
|  | \| *Anthurium fornicifolium* Croat \|  \| \| --- \| --- \| | **17** | **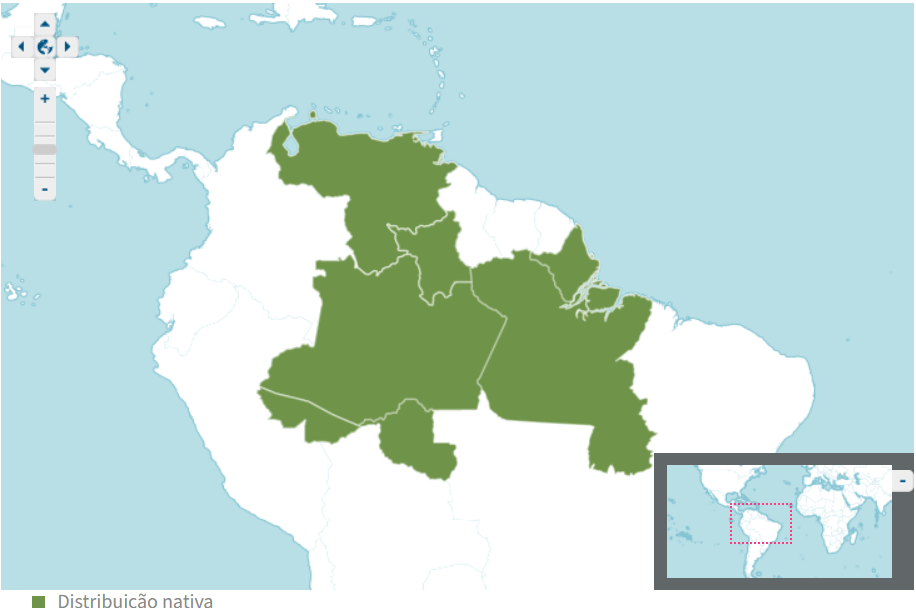** |
|  | *Anthurium holquianum* Croat & D.C.Bay | **18** | **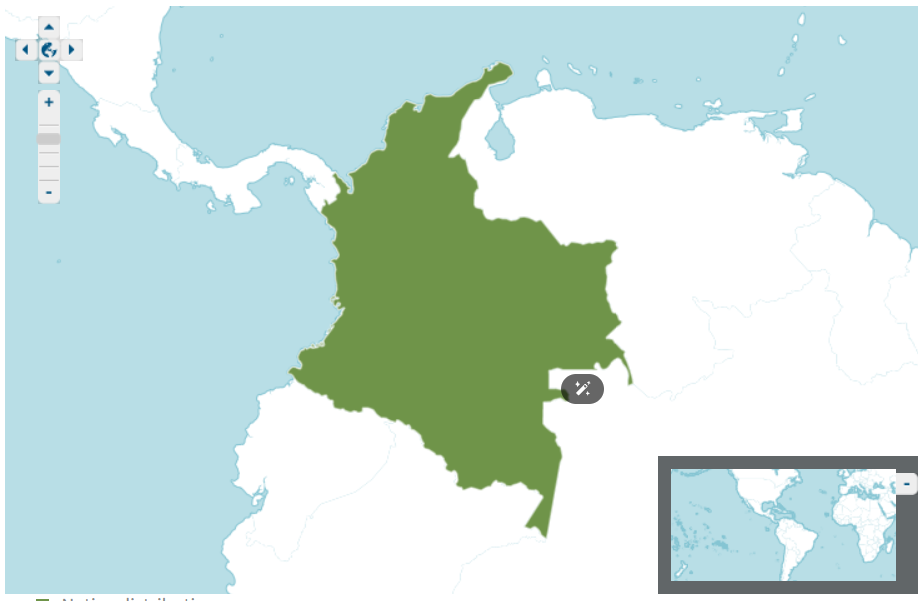** |
|  | \| *Anthurium krukovii* Croat \|  \| \| --- \| --- \| | **16** | **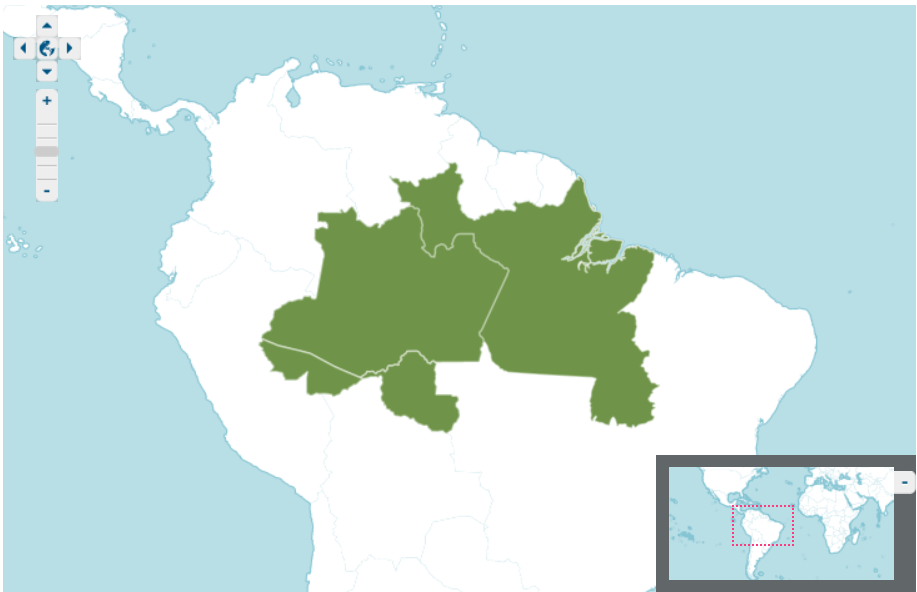** |
|  | \| *Anthurium latissimum* Engl*.* \|  \| \| --- \| --- \| | **28** | **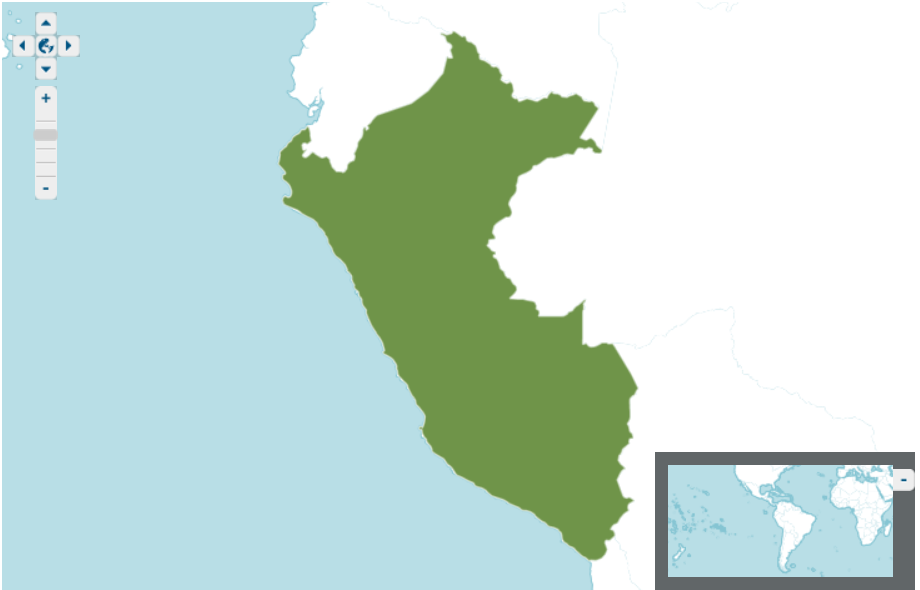** |
|  | \| *Anthurium llewellynii* Croat \|  \| \| --- \| --- \| | **18** | **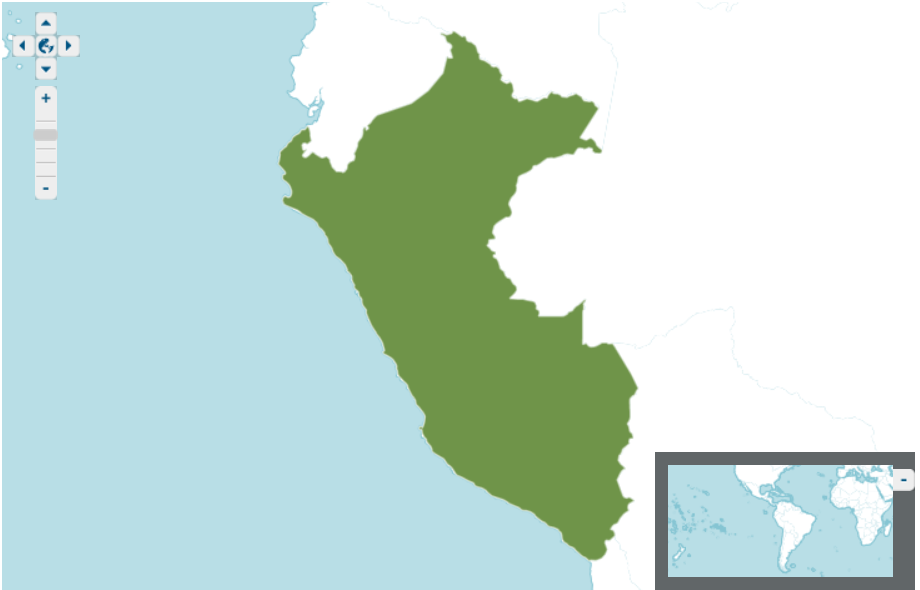** |
|  | \| *Anthurium moonenii* Croat & E.G.Gonç. \|  \| \| --- \| --- \| | **18** | **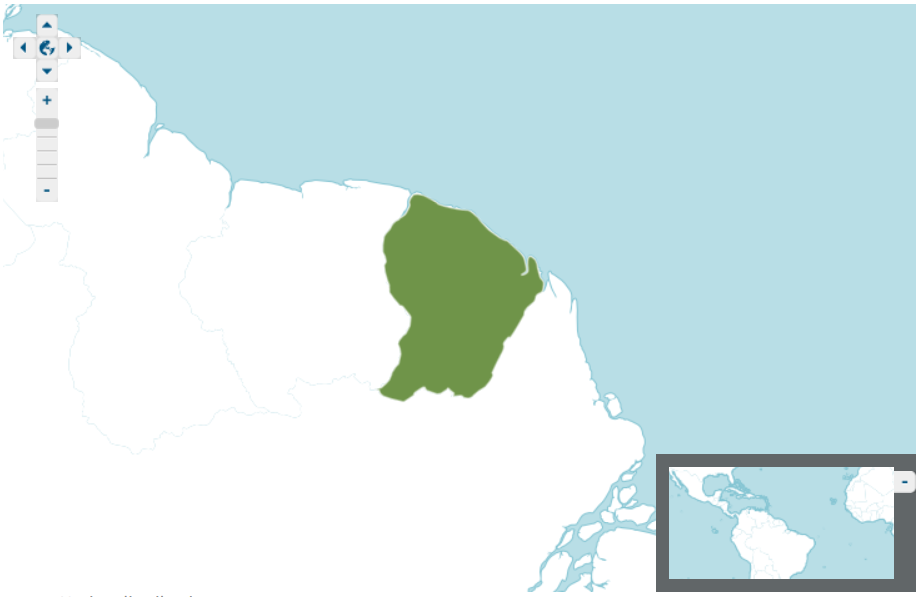** |
|  | \| *Anthurium stephanii* Croat & Acebey \|  \| \| --- \| --- \| | **28** | **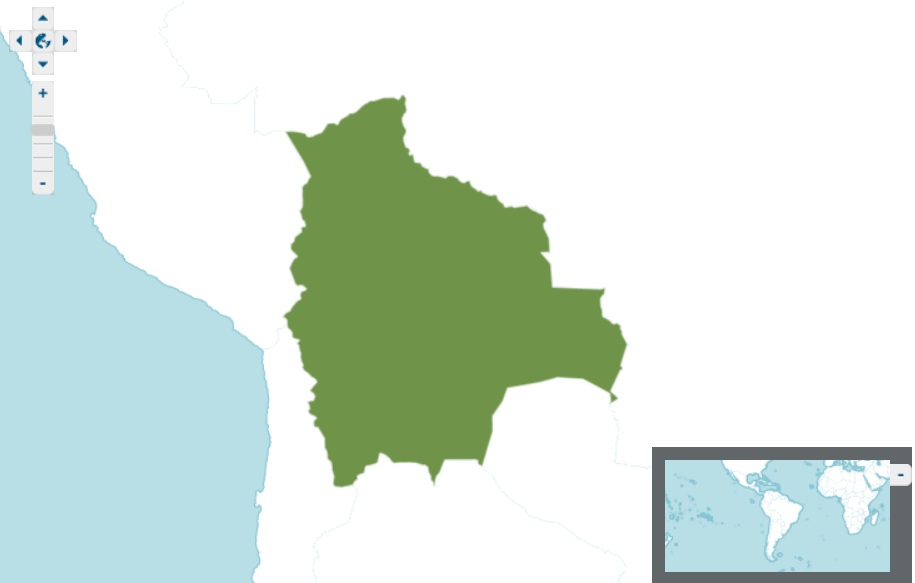** |
| **Bromeliaceae** | \| *Aechmea aquilega* (Salisb.) Griseb. \|  \| \| --- \| --- \| | **30** | **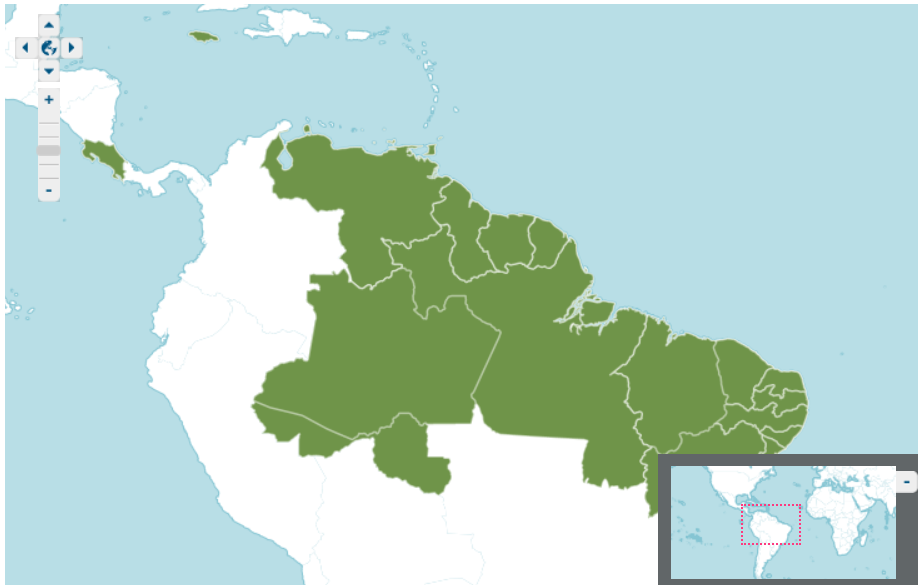** |
|  | \| *Aechmea fernandae* (E. Morren) Baker \|  \| \| --- \| --- \| | **43** | **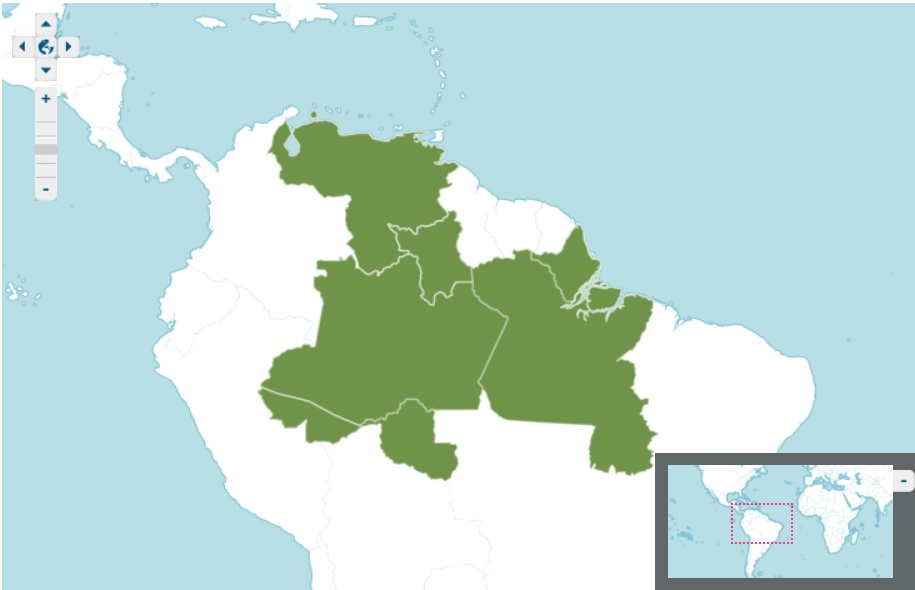** |
|  | *Aechmea rodriguesiana* (L.B.Sm.) L.B.Sm. | **27** | **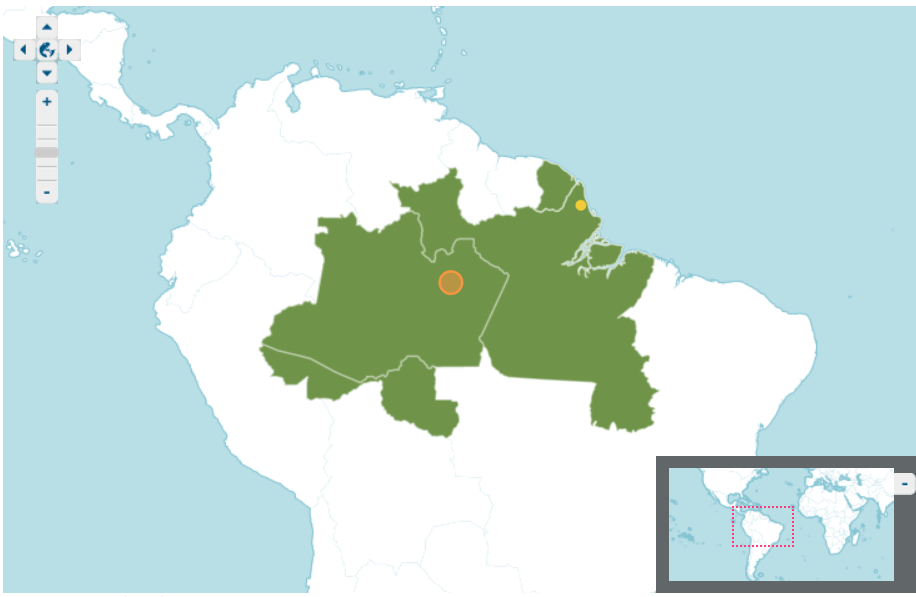** |
|  | \| *Guzmania vittata* (Mart. ex Schult. f.) Mez \|  \| \| --- \| --- \| | **40** | **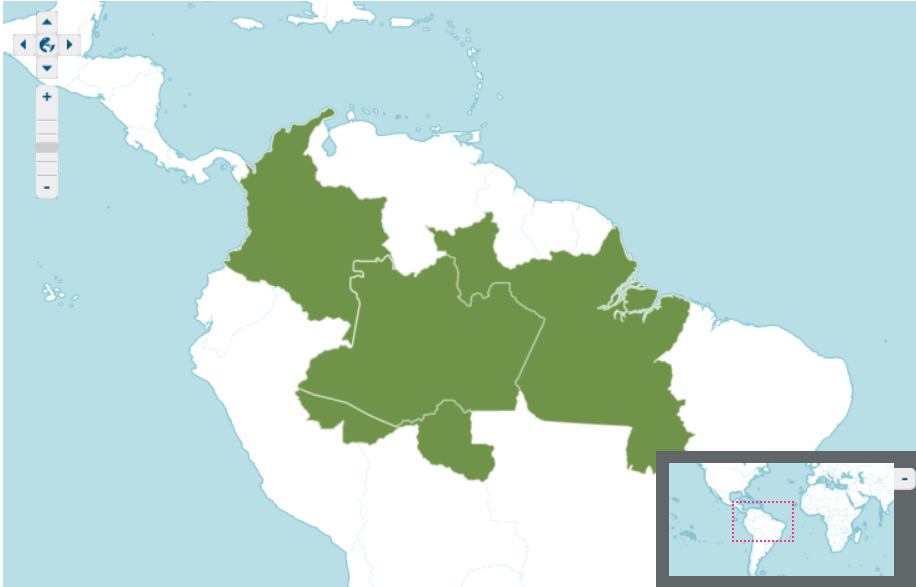** |
| **Ericaceae** | \| *Psammisia sclerantha* A.C. Sm. \|  \| \| --- \| --- \| | **55** | **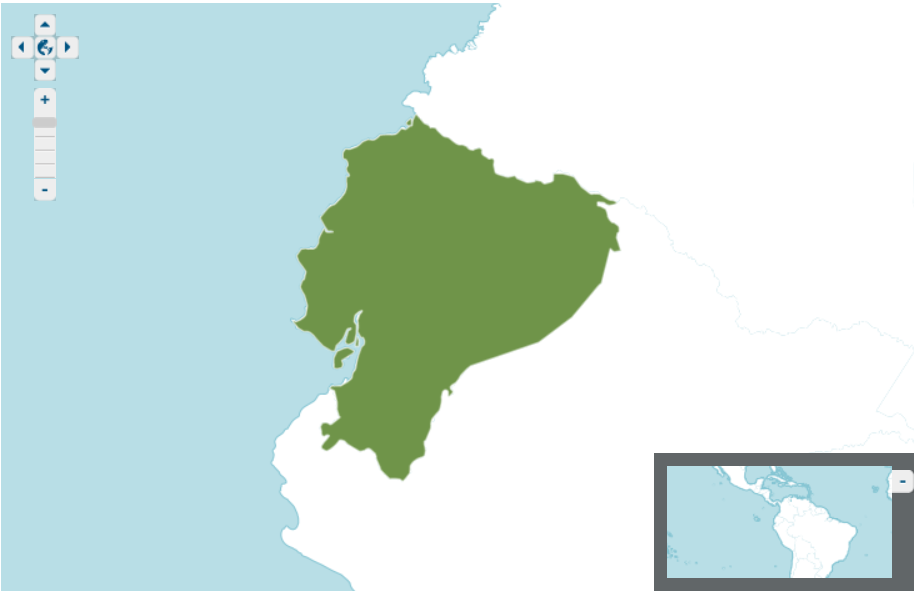** |
|  | \| *Themistoclesia orientalis* Luteyn \|  \| \| --- \| --- \| | **22** | **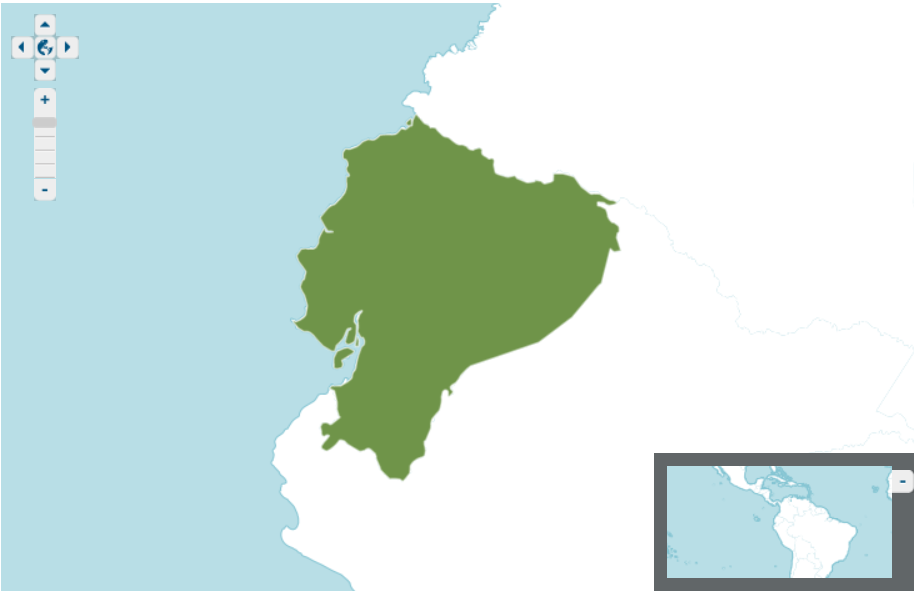** |
| **Melastomataceae** | \| *Miconia serpens* (Triana) Cogn. \|  \| \| --- \| --- \| | **54** | **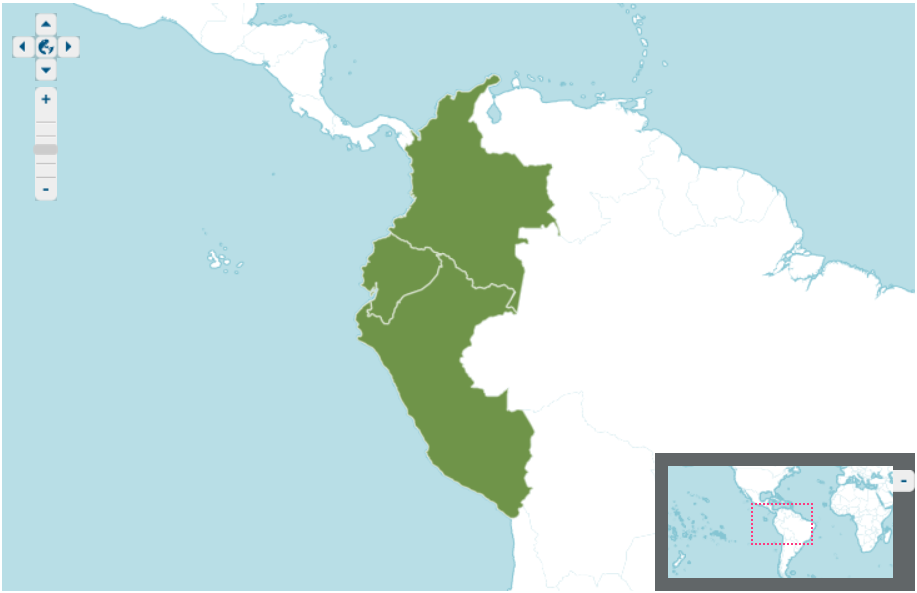** |
| **Orchidaceae** | \| *Catasetum hopkinsonianum* G.F. Carr & V.P. Castro \|  \| \| --- \| --- \| | **22** | **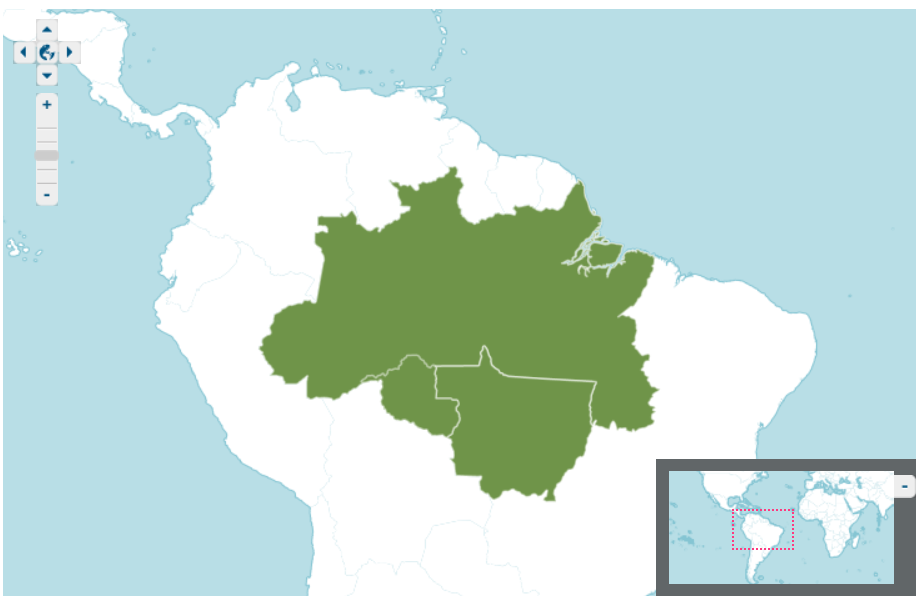** |
|  | \| *Catasetum tigrinum* Rchb. f. \|  \| \| --- \| --- \| | **32** | **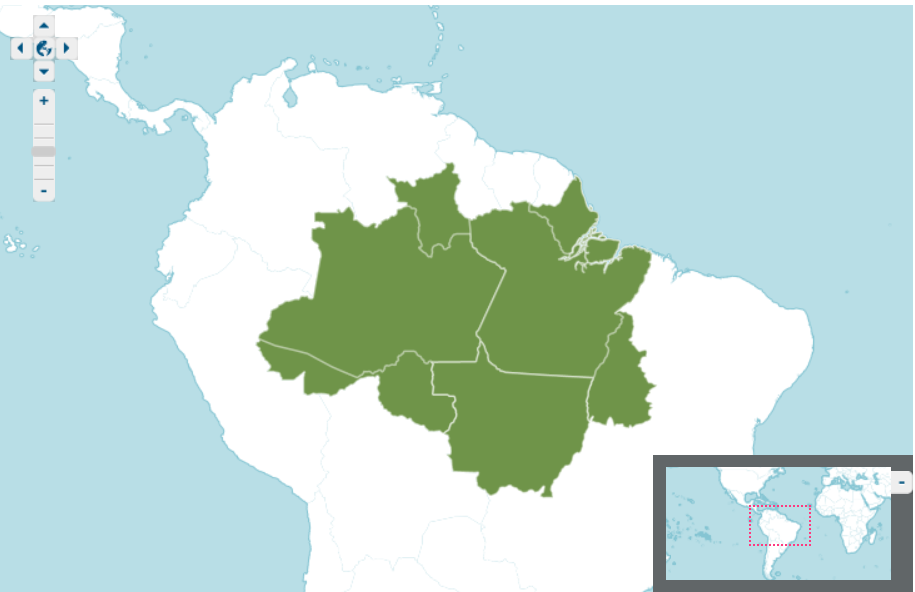** |
| **Piperaceae** | \| *Peperomia fluviatilis* Yunck. \|  \| \| --- \| --- \| | **25** | **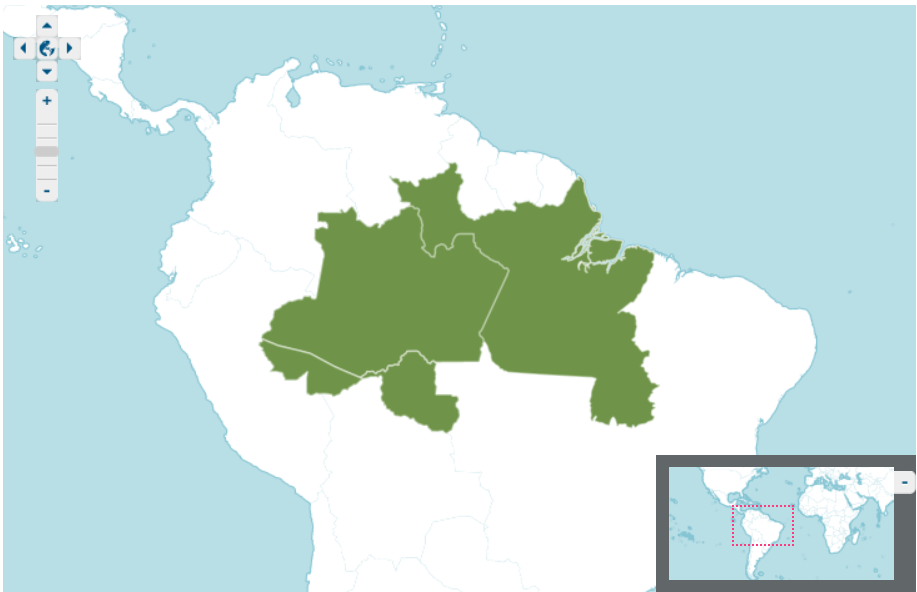** |
| **Urticaceae** | *Coussapoa sprucei* Mildbr. | **17** | **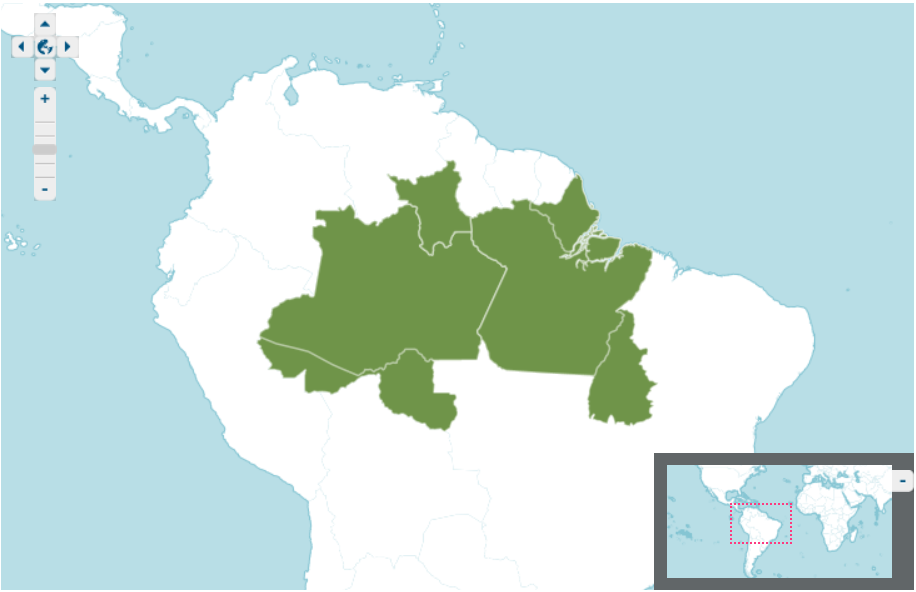** |

Real distribution occurrence
